# Dielectrophoretic Assembly of Customized Colloidal Trimers

**DOI:** 10.1021/acsnanoscienceau.5c00007

**Published:** 2025-03-12

**Authors:** Samira Munkaila, Kevin J. Torres, Jennifer Wang, Marcus Weck

**Affiliations:** Molecular Design Institute, Department of Chemistry, New York University, New York, New York 10003, United States

**Keywords:** dielectrophoresis, customized colloids, gold coating, surface anisotropy, colloidal assembly, colloidal trimers, microfabrication

## Abstract

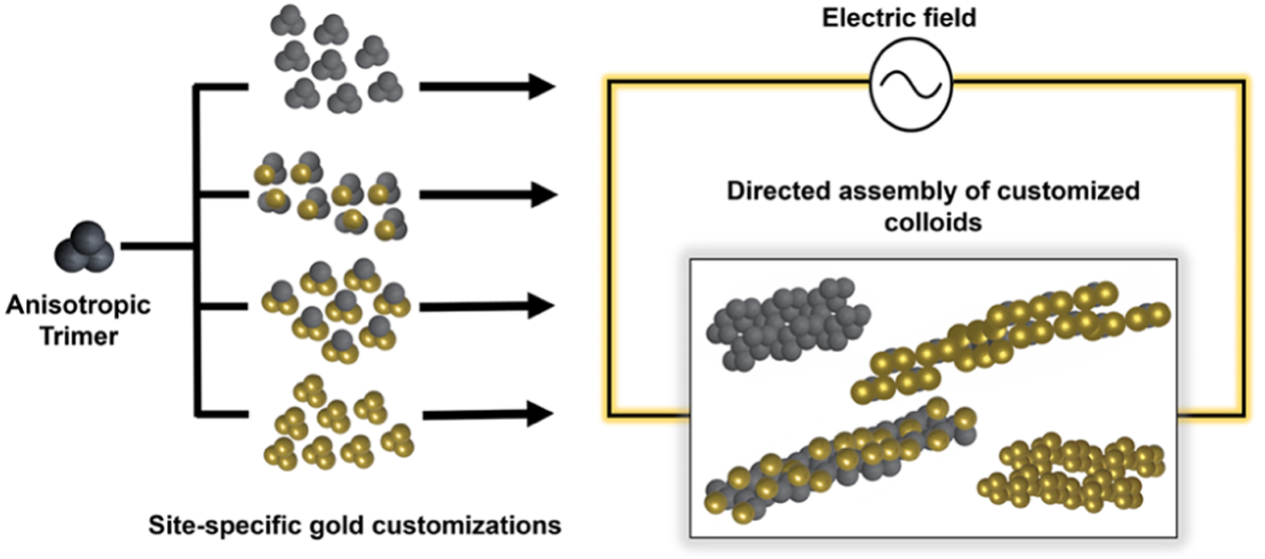

The controlled assembly of colloidal trimers with both shape and surface anisotropy remains a challenge. In this work, polymeric dielectric colloidal trimers selectively functionalized with gold nanoparticles are used to create four distinct particles. The shape and surface anisotropy provided by the metallodielectric particles allows for directive assembly in a dielectrophoretic field. When subjected to varied frequencies and media permittivities, the particles assemble with different packing densities and orientations. On-demand assembly and disassembly of the particles are achieved by switching on or off the applied voltage. These multicomponent colloidal particles and their subsequent assemblies presented here provide a promising platform for engineering complex structures with versatile functionalities.

## 1 Introduction

Colloidal particles have been used to create materials with an array of properties ranging from stimuli-responsiveness, self-healing, and liquid crystallinity^1,2^ and for various applications in energy storage, medicine, and advanced materials.^3,4^ The size, shape, and composition of the colloidal building blocks can be easily customized, allowing for the introduction of anisotropy.^5^ The synthesis and assembly of shape-tunable colloidal particles has opened an avenue for new colloidal assemblies.^6^ For example, colloidal dimers with precisely tunable symmetric and asymmetric shapes produce rich, structurally diverse assemblies with varying crystalline phases.^6^ As a result of the unique characteristics and advantages offered by both single and multiple-component colloidal assemblies, sophisticated design variations have been reported.^7^

Multicomponent colloidal particles, unlike colloidal dimers and clusters, are more challenging to synthesize.^8^ Multicomponent colloidal particle fabrication is typically achieved by sequential seeded growth where nucleation and growth conditions can be tuned.^5,8,9^ This synthetic route endows these particles with tailorable properties such as compositionally distinct functionality and controlled geometry, providing an avenue for complex colloidal assemblies. One such example is customizable chiral colloids^5^ with their unique chiroptical properties and potential for digital colloidal computation and programmable assembly.

Anisotropic trimer colloids, a subclass of multicomponent colloidal particles, have gained attention in recent years.^10^ Linear trimers with metallodielectric patches,^11,12^ nonlinear trimers with triangular patches and locomotive assembly behaviors,^13^ and “Mickey Mouse” colloids with patches that convey directional information^14^ have all been reported. These shape-anisotropies of the particles can alter the directionality of particle interactions during colloidal assemblies.

Another strategy for introducing anisotropy to a colloidal particle is via surface functionalization.^11^ This is achieved by localized functionalization, for example, with DNA,^15^ via various heterogeneous surface coatings that yield physically or chemically patterned Janus particles,^16^ or by incorporating metal coatings onto the selective surface(s) of particles.^17^ Gold nanoparticles, in particular, present high stability, variable optical properties, and tunable surface functionalization and can be synthesized in a straightforward fashion.^18−23^ By varying the surface chemistry of an isotropic colloidal particle to a gold-coated anisotropic particle, unique properties can be derived from both organic and metallic moieties for applications in plasmonics and optoelectronics.^24^ Further functionalization and customization of these anisotropic colloidal particles have not been explored due to several factors, including difficulty in scaling their synthesis, purification, and, most importantly, reproducibility.^25,26^ The lack of control over growth protocols for colloids with two or more distinct components poses a series of challenges for the desired multicomponent particle synthesis, hence the need for a facile synthetic process and a directed assembly protocol.^27,28^

The directed assembly of colloidal particles depends on the presence of regioselective interactions between the particles. Various strategies have been used for directed colloidal assembly, including physical, chemical, fluidic, and induced external field methods.^29−31^ Colloidal assembly via external electric fields allows for the formation of tailored architectures by tuning interparticle interactions through variations in field parameters.^32−34^ The use of dielectrophoresis (DEP) has expanded to the transport, separation, sorting, and assembly of different types of particles.^35−37^ With the ability to tune particle interactions on-demand by adjusting field parameters, DEP becomes viable for building complex structures such as microrobots and nanosensors for applications such as drug delivery and tissue engineering.^38−40^ More recently, linear metallodielectric particles were synthesized and assembled into various architectures in a DEP field.^41^ We rationalize that designing the shape and composition of multipatch particles, such as trimer particles with increased anisotropy by gold-coated customizations, opens the possibility of attaining particle assemblies with complex architectures for advanced materials.

**Figure 1:**
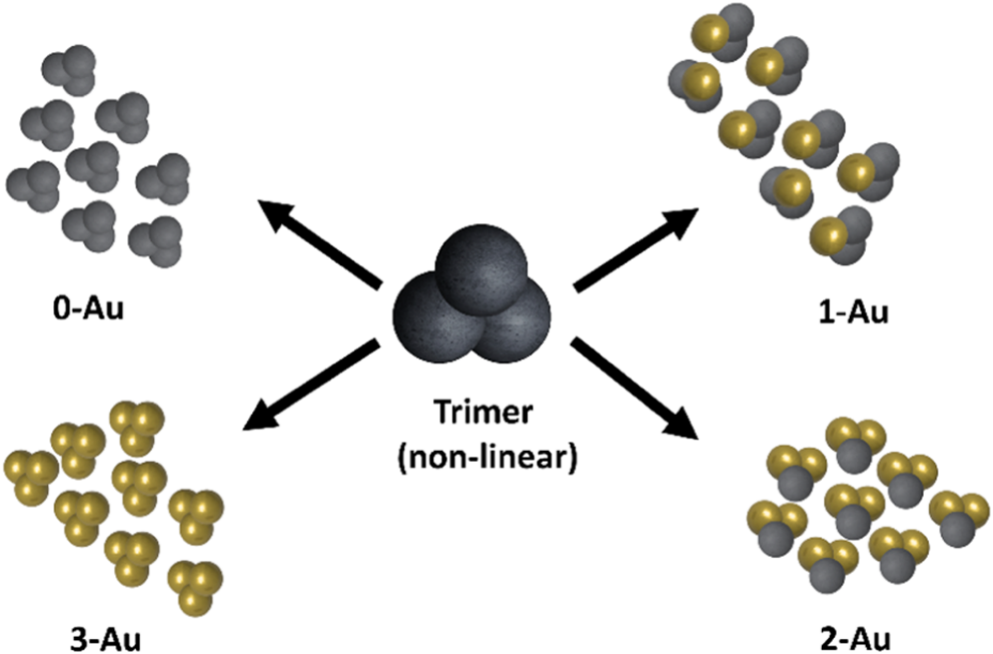
Schematic representation of the site-specific gold customization of trimer particles investigated in this study. The gray lobes represent the bare lobes, while the gold lobes represent the site-specific gold coating (for varying lobes) of each trimer particle.

In this contribution, we demonstrate a bottom-up technique for the sequential seeded growth synthesis of customized colloidal nonlinear particles with three lobes, the selective coating of individual lobes with gold, and the use of DEP to manipulate and direct the assembly of these particles. The tailored customization of each compartment includes the incorporation of a layer of gold nanoparticles on site-specific lobes (Figure 1), thereby changing the physicochemical properties of the lobe, their interaction with an electric field, and, ultimately, their directionality during assembly. We explore the assembly of various symmetrical lobe protrusions with different metallodielectric characteristics in deionized water (ε = 78.5) and aqueous potassium chloride (0.15 mM, ε = 49).^19,42,43^ The DEP force, F_DEP_, is proportional to Clausius–Mossotti (CM), which depends on the permittivity and conductivity of the particle (ε_p_, σ_p_) and medium (ε_m_, σ_m_). As the frequency of the applied electric field changes, the relative polarizability of the particle and the medium also changes due to frequency-dependent conductivity and dielectric relaxation effects.^42,43^ Therefore, increasing the gold coating on selected lobes enhances conductivity and asymmetric polarizability, shifting assembly from dielectric-dominated (**0-Au**) to metal-dominated (**3-Au**).

Gold nanoparticles are uniquely suited for DEP studies due to their exceptional electrical polarizability, chemical stability, and tunable surface functionalization. The high conductivity and permittivity contrast of gold relative to polymeric particles (ε_Au_ = 6.90, ε_TPM_ = 6.58) (σ_Au_ = 4.5 × 10^7^ S/m, σ_TPM_ = 1.0 × 10^–15^ S/m)^36,44^ creates strong, localized DEP forces under AC fields, enabling precise control over particle orientation and assembly. By selectively coating the lobes of trimer particles with gold, we systematically modulate the spatial distribution of conductivity and permittivity gradients. This design allows us to isolate the effects of dielectrophoretic forces, dipole–dipole interactions, and van der Waals (vdW) adhesion in a controlled manner. An electrolyte with a significantly lower dielectric constant, like potassium chloride, causes a decrease in the distance between particle surfaces due to electrostatic interactions.^36,42,44^ Our study elucidates the DEP assemblies of colloidal trimers by varying the frequency of the electric field and the permittivity of the surrounding medium.

## 2 Experimental Section

### 2.1 Synthesis of Colloidal Trimers

**Figure 2:**
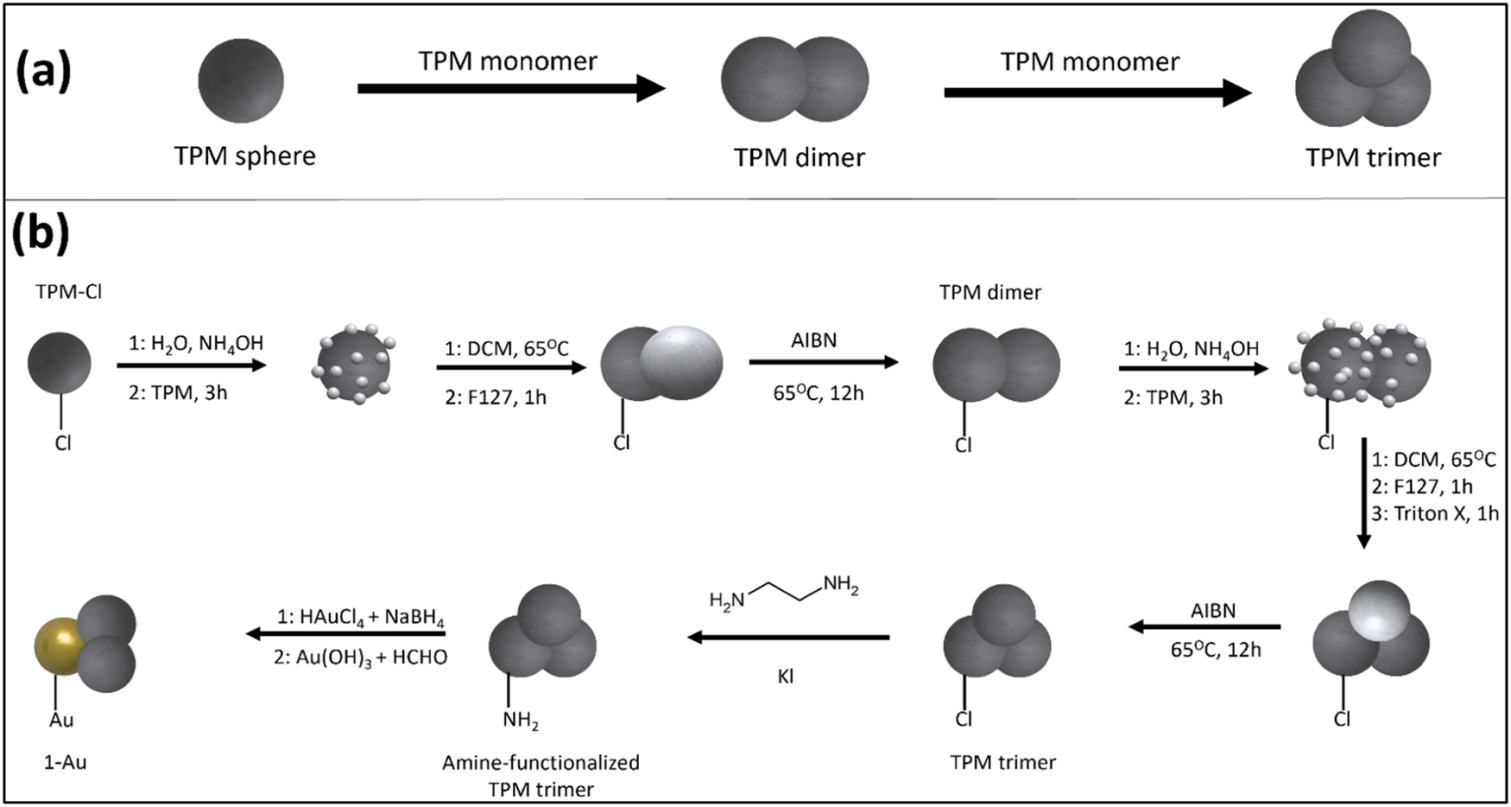
(a) Simplified illustration of the sequential seeded growth process for the synthesis of the bare trimers (b) detailed schematic representation of trimer particle synthesis and gold coating with the **1-Au** synthesis as the example: light gray lobes show hydrolyzed TPM and dark gray lobes show polymerized TPM particles.

The synthesis of the trimers is described in Figure 2 and was achieved by a sequential seeded growth methodology. In detail, monodispersed, micron-sized colloidal spheres were first formed via spontaneous emulsification using a base-catalyzed hydrolysis and condensation procedure,^45^ which has been used in the literature to produce spherical as well as anisotropic particles.^46−48^

Each lobe of the particles was synthesized from 3-(trimethoxysilyl)propyl methacrylate (TPM) (Figure 2a). The −Si (OMe)_3_ groups can be hydrolyzed and subsequently converted to cross-linked poly(silsesquioxane)s by base-catalyzed polycondensation.^45,49,50^ To incorporate chlorine functionalities on select lobes, 3-chloro-2-hydroxypropyl methacrylate (CHPMA) was added and allowed to diffuse into the TPM emulsion. Pluronic F127 surfactant was incorporated into the particle surfaces to tune surface wetting and stabilize the colloidal dispersion. Subsequently, upon the addition of the free radical initiator 2,2′-azobis(2-methylpropionitrile) (AIBN), the liquid (monomer) droplets were polymerized via free radical polymerization of the acrylates to produce solid TPM spheres.

**Figure 3:**
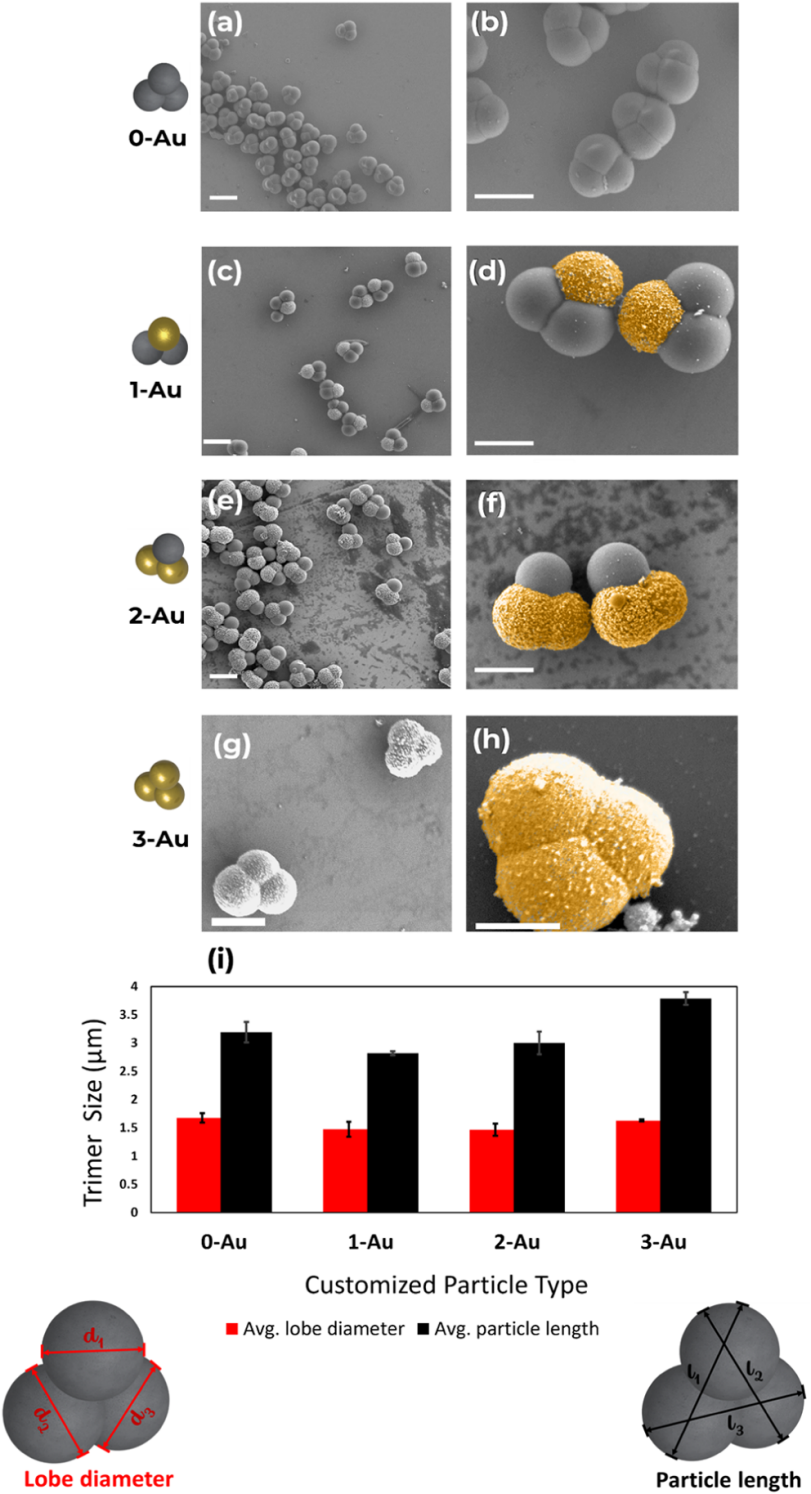
(left) Schematic representation of all four nonlinear trimers **0-Au**, **1-Au**, **2-Au**, and **3-Au**. (a, b) Scanning electron microscopy (SEM) images of **0-Au** trimer particles. (c, d) SEM images of **1-Au** trimer particles with one gold lobe. (e, f) SEM images of **2-Au** trimer particles with two gold lobes. (g, h) SEM images of **3-Au** trimer particles with three gold lobes. The gold-coated component has a brighter contrast under the SEM. (i) Size distribution of the customized trimers with corresponding schematics defining the lobe diameter and lobe length for the trimer particles. Gold lobes in (d, f, h) have been falsely colored for clarity. Scale bars, 2 μm.

Serving as seeds for the dimer particles, the TPM solid spheres were anchored in randomly distributed TPM oligomer droplets, thus nucleating TPM oligomers on the surface of each seed particle via base-catalyzed hydrolysis. The seeded growth steps were performed in glass vials on a rotating station. Upon the addition of dichloromethane (DCM), these droplets coalesce to form a single lobe. Next, the suspension was mildly agitated to ensure complete merging of the droplets into a single lobe protrusion on each seed particle, followed by complete evaporation of the DCM (Figure 2b). Pluronics F127 (1.0 wt % aqueous solution) was added to the suspension to adjust the interfacial tension of the final dimer. After polymerization with AIBN, dimers with dumbbell morphology were formed. A third lobe was added to the dimer particle by a base-catalyzed oligomerization of the TPM monomer onto the dimer-seed particle via a process similar to that described above. An extended hydrolysis time during the trimer synthesis led to an undesired linear trimer morphology due to a decrease in interfacial energy between the water and the seed phase (which promoted the spread of TPM on the seed surface)^6^ (Figure S2). The addition of Triton X-100 to the trimer suspension, however, induced dewetting of the coalesced oligomer lobe on the seed surface, leading to an evenly protruding lobe with nonlinear morphology. Triton X-100 preferentially adsorbs onto the surface of the lobes and causes a notable change in contact angle.^51^ AIBN was then added to the mixture to polymerize the final lobe of the trimer particles. The resulting trimers were retrieved and washed with deionized (DI) water, centrifuged, and redispersed in DI water. The obtained trimers were washed and purified from secondary nucleation by density gradient centrifugation to obtain monodisperse bare trimers, **0-Au**, as shown in Figure 3a, and chlorine-functionalized trimers, respectively. For further details, see the Methods section.

### 2.2 Site-Specific Gold Coating and Characterization

The customization of each trimer particle with gold-coated lobes was achieved according to an established protocol^22^ to obtain gold-functionalized particles **1-Au** (one lobe coated with gold), **2-Au** (two lobes coated with gold), and **3-Au** (all lobes coated with gold), as shown in Figure 3. To initiate the gold coating, the chlorine groups on the surface of the chlorinated TPM particles were converted to amines by reaction with ethylenediamine. Then, gold was nucleated at the surface of the amine-functionalized particle via the formation of metal-ammine complexes using a modified literature procedure.^52^ Gold chloride was used as the precursor and treated with sodium borohydride (NaBH_4_), which reduces Au (III) to Au(0).^18^ The gold nanoparticles on the colloid surface template the growth of a gold layer in the presence of Au(OH)_3_ and formaldehyde. The thickness of the gold layer can be tuned by the concentration of the gold precursor and by varying the amounts of NaBH_4_ (Figure S4). Trimer particles with one gold lobe customization, **1-Au**, as shown in Figure 2b, were generated by using the previously described procedure on trimers in which only 1 lobe had chloride functionalization (TPM-Cl dimer), followed by selectively coating the specific lobe using the protocol described above. Particles **2-Au** and **3-Au**, with two and three gold-coated lobes, respectively, were synthesized analogous to 2 or 3 chlorine-functionalized lobes of the trimers.

## 3 Results and Discussion

Scanning electron microscopy (SEM) was used to image the trimers and identify the topography of the customized particles. Ten of each particle type were measured randomly using the SEM analysis tool. The statistics for the particles in Figure 3i show the average lobe diameter and length representing the distance between adjacent lobes.

Each trimer with a gold-coated lobe appears brighter in the SEM micrograph due to gold’s higher conductivity compared to TPM.^41^ Energy-dispersive spectroscopy (EDS) was used to quantify the distribution of gold nanoparticles on the site-specific lobe. Mapping confirms that about 80 wt % of gold nanoparticles uniformly coated the customized surface (Figure S1C). For further details, see the Experimental section in the Supporting Information.

### 3.1 DEP Assemblies of Customized Trimer Particles

**Figure 4:**
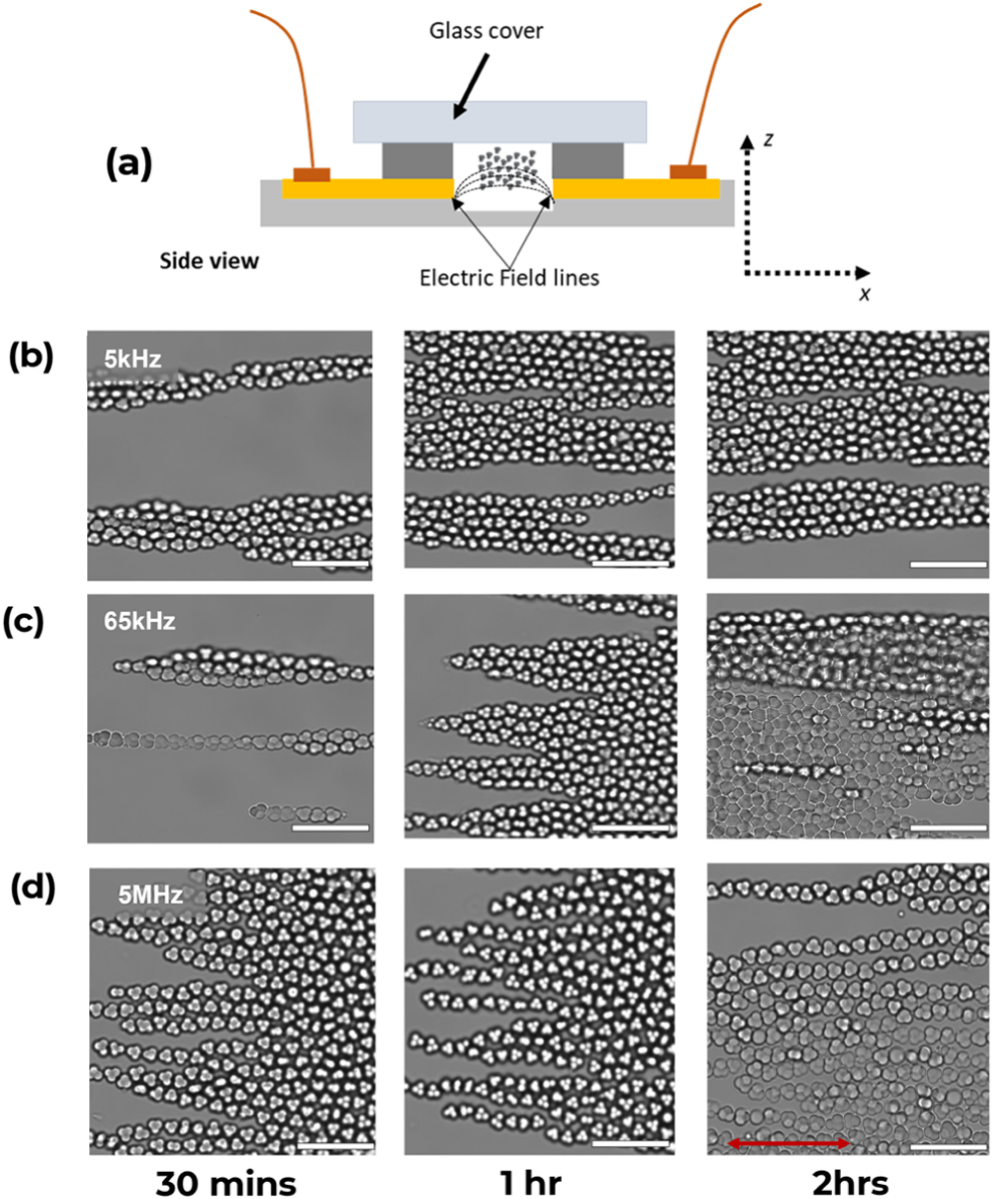
Optical micrographs of **0-Au** assembled in deionized water (ε = 78.5) under DEP force with increasing frequency and constant electric field intensity, *E* = 200 Vcm^–1^. (a) Schematic illustration of the side view of the DEP device containing **0-Au**. (b) Trimer particle assembly at *f* = 5 kHz. (c) Trimer particle assembly at *f* = 65 kHz. (d) Trimer particle assembly at *f* = 5 MHz. Scale bars: 10 μm. The red arrow indicates the direction of the electric field.

Next, we investigated the assembly of the particles in a DEP field. For these studies, the customized multicomponent particles were purified by density gradient centrifugation to obtain monodisperse trimer particles. The monodisperse particles were suspended in deionized water, vortexed, and then injected into the channel of the DEP device (Figure 4a). Particles were subjected to DEP forces based on variations in the frequency of the AC electric field, permittivity of the surrounding media, and dielectric constants of the particle.^44^ The assembly of the particles in the DEP field was captured with a bright-field optical microscope. To initially examine the effect of the frequency on the packing orientation of each customized trimer particle, we analyzed the various assemblies with varying frequencies and constant electric field intensity. An electric field intensity, *E*, of 200 Vcm^–1^ and frequencies, *f*, between 10 kHz and 5 MHz were used to induce self-assembly. In addition to frequency variation, the media in which the particles were suspended was also varied: deionized water with a dielectric constant, ε, of 78.5 and aqueous potassium chloride, 0.15 mM KCl (ε = 49).^19,42,43^ Solutions’ permittivity affects the force experienced by each particle in a DEP field.^36,57^ The polarization caused by the applied AC field oriented the dipole moments of the particles under ambient conditions. Figure 4 shows the optical micrograph of the assemblies of **0-Au** particles in deionized water under DEP over 2 h.

**Figure 5:**
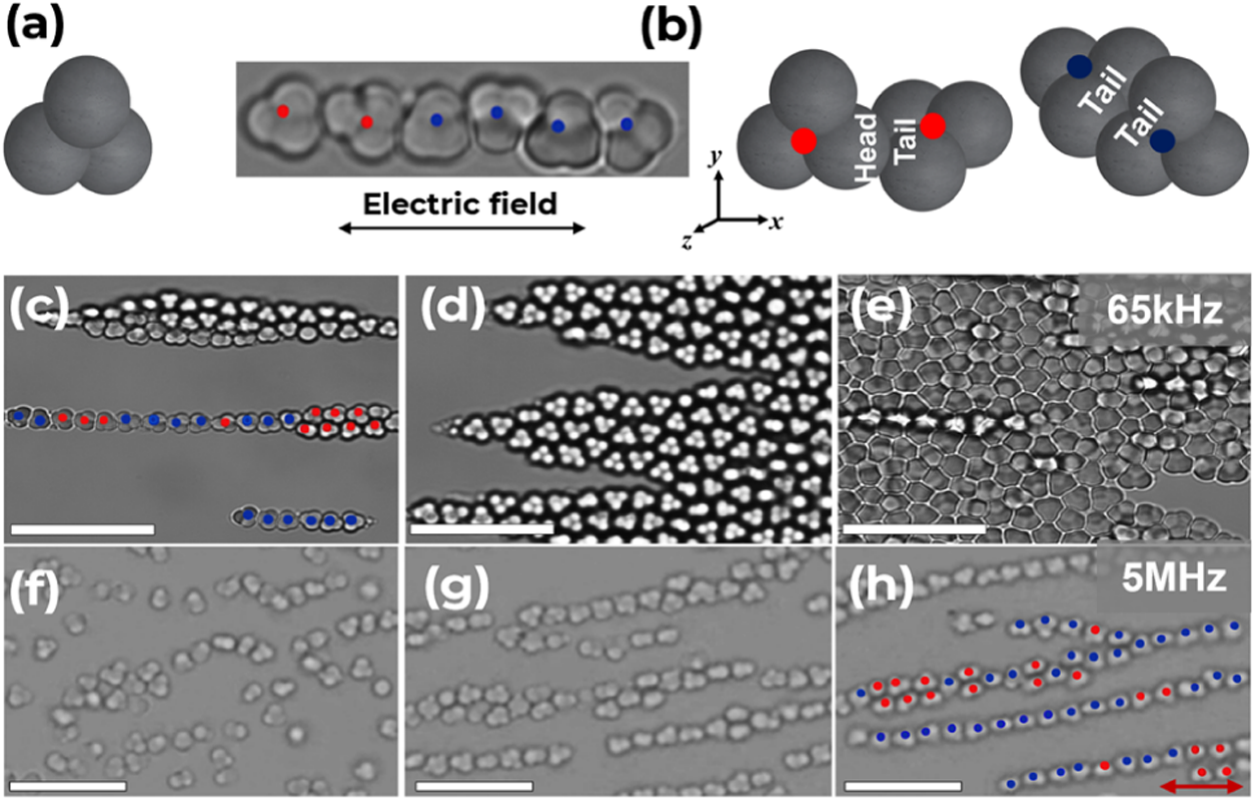
(a) Schematic representation of bare trimers with no gold lobes, **0-Au**. (b) Excerpt from the DEP assembly of **0-Au** and corresponding schematic representation of “head-to-tail” and “tail-to-tail” assembly orientations for **0-Au**. (c, d, and e) Optical micrographs of DEP assembly of **0-Au** in deionized water (ε = 78.5) at *f* = 65 kHz from 30 min to 2 h, respectively. (f, g, and h) Optical micrographs of DEP assembly of **0-Au** in KCl (ε = 49) at *f* = 5 MHz from 30 min to 2 h, respectively. Electric field intensity of *E* = 200 Vcm^–1^. Red dots show head-to-tail orientations, while blue dots show tail-to-tail orientations. Scale bars = 10 μm. The red arrow indicates the direction of the electric field.

The dielectric constant of the bare trimer particles, ε, is 2.8,^41^ which is significantly lower than that of the surrounding aqueous medium, making the self-assembly concentrate away from the region of maximum field strength, displaying negative DEP (*n*-DEP) behavior upon introducing the AC electric field.^36^Figure 5 shows the optical micrograph of assemblies of **0-Au** particles in deionized water and KCl, respectively, under DEP. At a frequency of 5 kHz, linear chains of **0-Au** trimer particles, where none of the lobes are functionalized, initially aligned with no preferred individual particle orientation to the direction of the electric field due to their 3-fold rotational symmetry about a central axis, which is perpendicular to the applied electric field. We therefore attribute the “head-to-tail” and “tail-to-tail” orientation to denote the relationship of a particle with respect to its neighboring particle during assembly.

The **0-Au** particles with only one lobe in contact with multiple lobes of a neighboring **0-Au** particle formed the head-to-tail orientation, whereas those with more than one lobe in contact with multiple lobes of a neighboring particle formed tail-to-tail assemblies, as shown in Figure 5b. Upon increasing the frequency to 65 kHz, the linear chains aggregated further, yielding denser packing and decreased lattice spacings. After 2 h, the particles experienced the highest stacked 2-D packing density, as shown in Figure 5. Further increase of the frequency to 5 MHz led **0-Au** to oscillate along the field direction with a corresponding decrease in packing density and concentrated along the gradient toward the region of maximum field strength, positive DEP (*p*-DEP), shown in Figure 4d. These observations suggest that **0-Au** experienced a “crossover frequency” – the frequency at which the particles and medium are equally polarizable, resulting in no net dielectrophoretic force.^53^Figure 5h shows the formation of a cascade of 2-D chains of **0-Au** in KCl at a higher frequency of 5 MHz and relatively fewer chains at a lower frequency of 65 kHz (Figure S5B).

**Figure 6:**
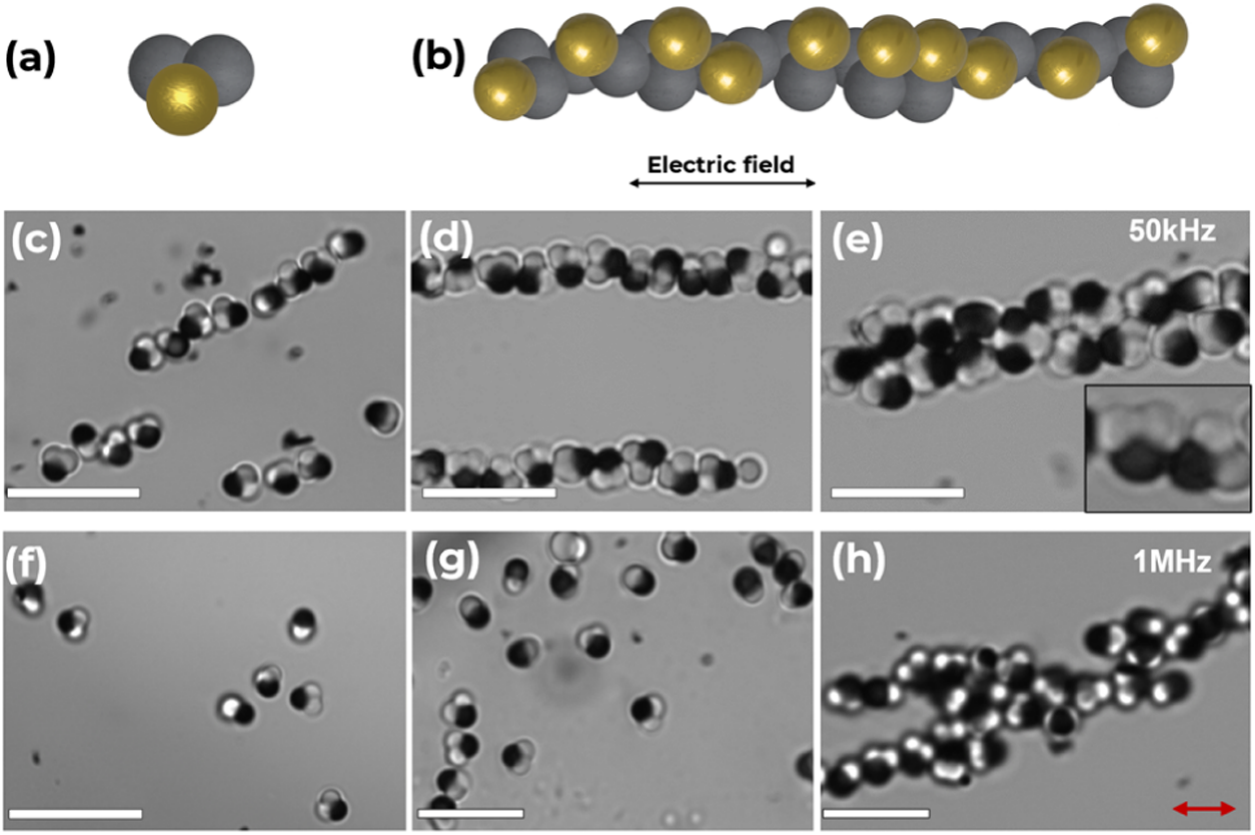
(a) Schematic representation of nonlinear trimer with one gold lobe, **1-Au**. (b) Excerpt from the DEP assembly of **1-Au** and corresponding schematic representation of the assembly orientation. (c, d, and e) Optical micrographs of DEP assembly of **1-Au** in deionized water (ε = 78.5) at *f* = 50 kHz from 30 min to 2 h, respectively. (f, g, and h) Optical micrographs of DEP assembly of **1-Au** in KCl (ε = 49) at *f* = 1 MHz from 30 min to 2 h, respectively. Electric field intensity of *E* = 200 Vcm^–1^. (e) Insert: Optical micrograph of two **1-Au** trimer particles under DEP. The gold-coated component has a darker contrast under a bright-field optical microscope. Scale bars = 10 μm. The red arrow indicates the direction of the electric field.

In response to AC fields, particles **1-Au**, shown in Figure 6, demonstrated assemblies distinct from those of **0-Au**. Unlike the assemblies of **0-Au**, the assemblies of **1-Au** start to form at lower frequencies. This could largely be attributed to the significantly higher conductivity of the gold-coated metallic portion of the metallodielectric **1-Au** trimer particle.^24^ When a frequency of 50 kHz was applied, **1-Au** particles formed lateral chains along the field lines resulting from the dipole–dipole interactions by the chaining force shown in Figure 6c.^42^ The gold-coated lobe dominated the assembly of **1-Au** by resting on the lower surface of the DEP cell, where the electric field is concentrated, thus orienting the particles in the *z-*direction.^41^ We attribute this behavior to the larger induced dipole resulting from the polarizability of the electrically neutral lobes of the trimer and the gold lobe, which have a higher conductivity. The bulk dielectric part of the particle formed a tail-to-tail orientation largely due to the comparable permittivity and lobe position of the bare TPM lobes. Its motion is also displayed in Supporting Movie S2. At a higher frequency of 1 MHz, the different lateral chains formed a dense lateral packing with each gold-coated lobe still maintaining its initial *z*-direction, forming staggered aggregates while upholding contact with neighboring gold lobes and the bare TPM lobes continuing contact with only similar lobes shown in Figure 6e,h.

**Figure 7:**
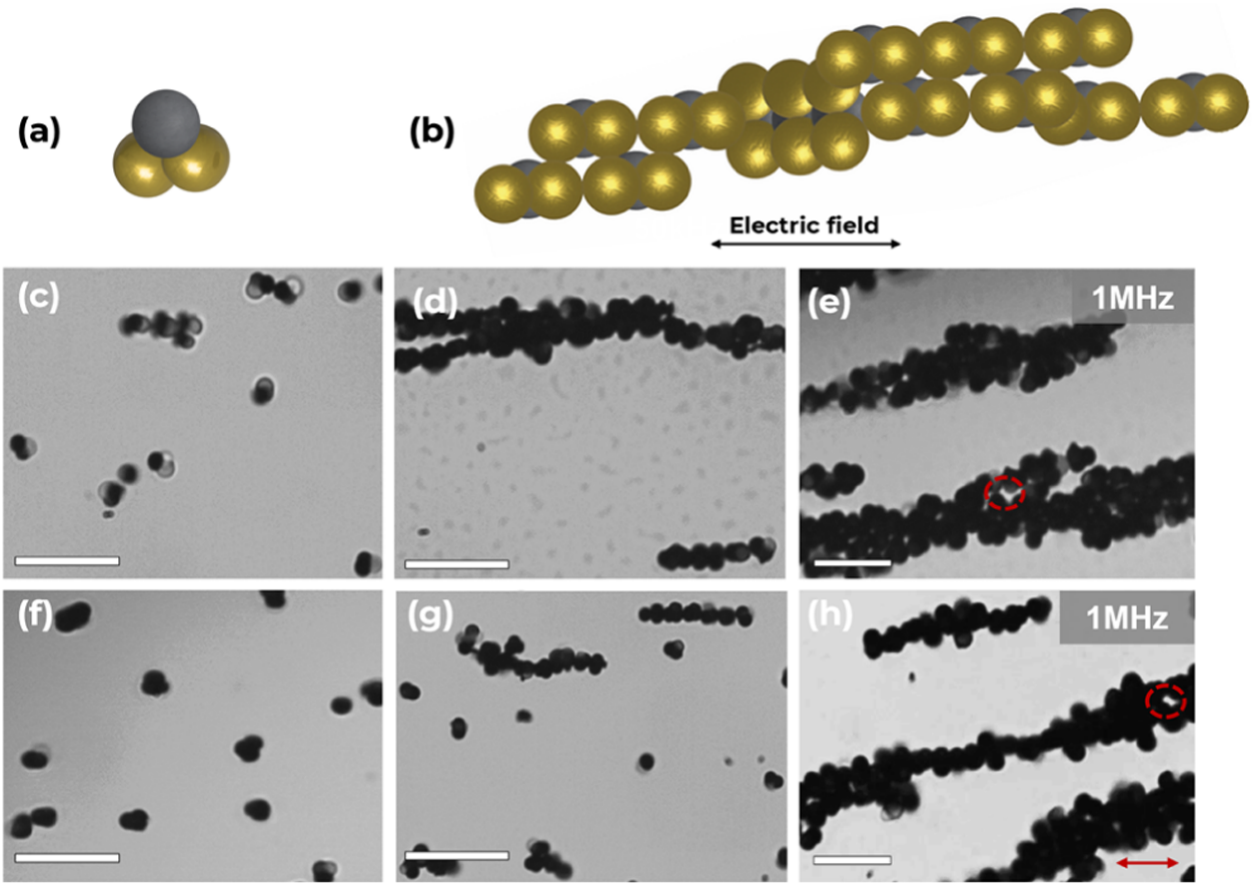
(a) Schematic representation of nonlinear trimer with two gold lobes gold customizations, **2-Au**. (b) Excerpt from the DEP assembly of **2-Au** and corresponding schematic representation of the assembly orientation. (c, d, e) Optical micrographs of DEP assembly of **2-Au** in deionized water (ε = 78.5) at *f* = 1 MHz from 30 min to 2 h, respectively. (f, g, h) Optical micrographs of DEP assembly of **2-Au** in KCl (ε = 49) at *f* = 1 MHz from 30 min to 2 h, respectively. Electric field intensity of *E* = 200 Vcm^–1^. The gold-coated component has a darker contrast under the bright field optical microscope. Scale bars = 10 μm. The red arrow indicates the direction of the electric field, and the red dotted circles show the “porous gaps”.

When **2-Au** particles were introduced to the AC electric field under DEP force, the two gold-coated lobes (with higher conductivity, σ, than TPM) governed the assembly orientation of each unit particle (ε_Au_ = 6.90, ε_TPM_ = 6.58) (σ_Au_ = 4.5 × 10^7^ S/m, σ_TPM_ = 1.0 × 10^–15^ S/m).^36,44^ At lower frequencies of 50 kHz, **2-Au** was observed to “rotate and flip” while forming staggered chains (Supporting Movie S3), which eventually cascaded into 2-D bundles at a higher frequency of 1 MHz. This assembly is medium-independent and has been observed for both deionized water and KCl, respectively, as shown in Figure 7d,g. Every staggered chain contains particles that were oriented so that their gold-coated lobes aligned with the metallic counterpart throughout the assembly, while their bare TPM lobes faced the reverse direction without contacting another particle. Regardless of the starting orientation, **2-Au** assembles into multiple staggered chains of gold-coated aggregates when the electric field is turned on, ascertaining that the DEP particle–particle interactions dominate the random Brownian motion originally possessed by the particles in the DEP device.^6^

After 2 h, the staggered chains of **2-Au** particles gradually aligned together while anchoring each chain via the two gold lobes on each particle. This led to the formation of “porous gaps” between each chain aggregate (Figure 7e,h). This preferential “open-structure” arrangement of the **2-Au** particles at higher frequencies can be attributed to the fact that the free rotation of metallodielectric particles about the axis parallel to the field lines influenced the interparticle interactions between the gold lobes (Supporting Movie S3). At all frequencies investigated, the metal particles were more polarizable than the assembly media due to their high conductivities.^54^ So, unlike dielectric particles, they preferred to migrate to regions of high field gradients experiencing a positive DEP *(p*-DEP).^36,44^

**Figure 8:**
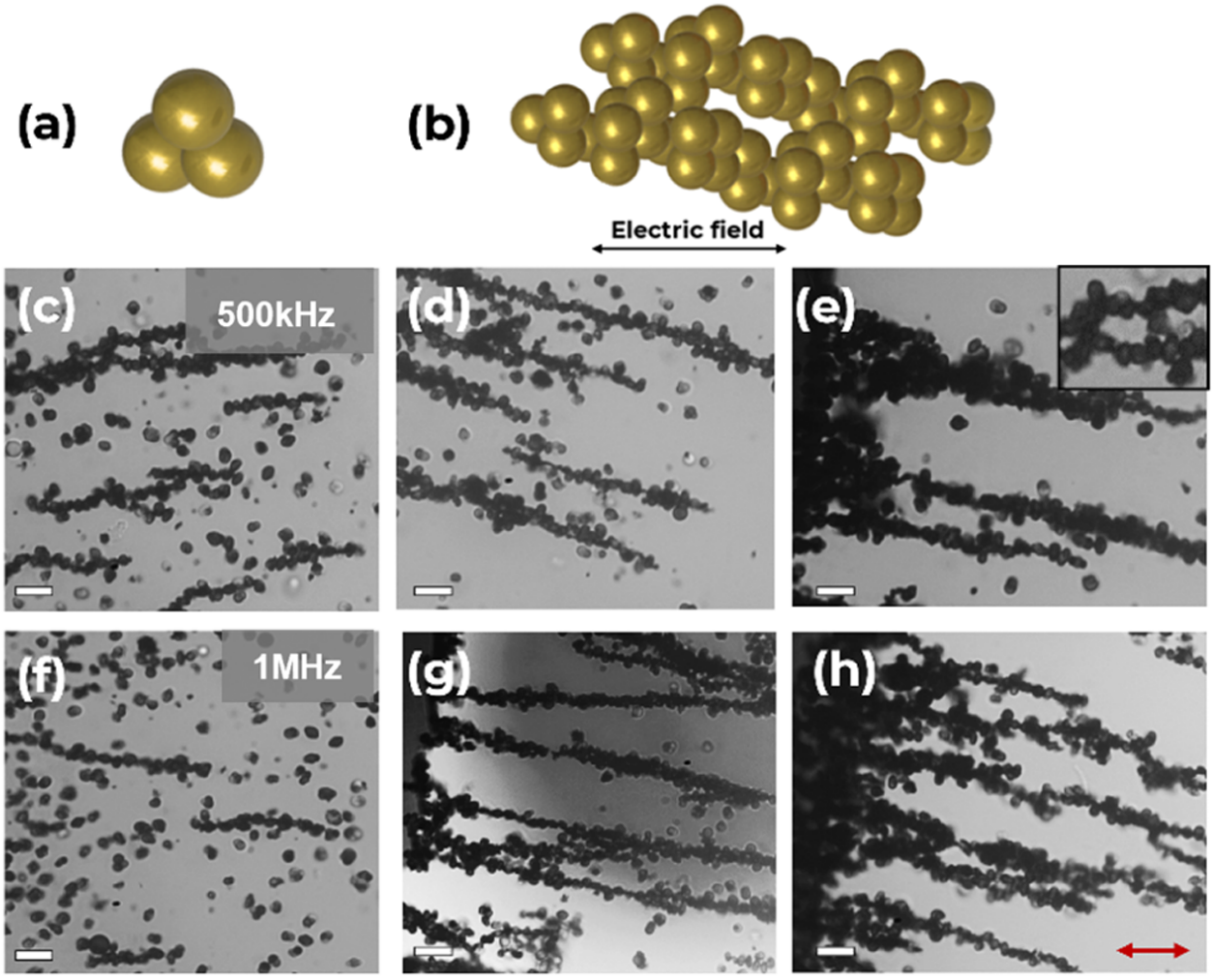
(a) Schematic representation of nonlinear trimer with three gold lobes, **3-Au**. (b) Corresponding schematic representation of the assembly orientation. (c, d, e) Optical micrographs of DEP assembly of **3-Au** trimer particles in deionized water (ε = 78.5) at *f* = 500 kHz from 30 min to 2 h, respectively. (f, g, h) Optical micrographs of DEP assembly of **3-Au** trimer particles in KCl (ε = 49) at *f* = 1 MHz from 30 min to 2 h, respectively. Electric field intensity of *E* = 200 Vcm^–1^. (e) Insert: Optical micrograph of a **3-Au** trimer particle network under DEP. The gold-coated trimers have a darker contrast under a bright field optical microscope. Scale bars = 10 μm. The red arrow indicates the direction of the electric field.

The **3-Au** particles exhibit the most metallic character and demonstrated an overall higher permittivity, allowing electrical energy to be retained briefly even after being disconnected from the AC source and upon varying electric field intensity of 100 Vcm^–1^ and 200 Vcm^–1^.^23,44^ The frequency dependence of the particle assembly of **3-Au** can be seen in Figure 8 for both deionized water and KCl, respectively, over a period of 2 h. The **3-Au** particle assemblies aligned with the electric field direction at the electrodes of the device are shown in Figure 8e,g,h. With an overall higher conductivity, these particles start aligning into chains at a frequency of 500 kHz. When a frequency of 1 MHz is applied, the DEP force becomes strong enough to overcome the low repulsive interactions experienced by the particles, causing additional particles to agglomerate toward the region of high field gradient, thus experiencing a positive DEP. Although **3-Au** were well dispersed in each medium at the start of the experiments, the repulsive interactions between each particle were not strong enough to prevent agglomeration over time. This can be attributed to the low ζ potential values of −11.7 and −35.2 mV in KCl and deionized water, respectively (Figure S6). By slowly increasing the electric field intensity from 100 Vcm^–1^ to 200 Vcm^–1^, **3-Au** formed cascading chains suggesting the “threshold voltage” where the minimum DEP force required for the formation of cascading aggregates overcomes the repulsive interparticle forces that stabilize the dispersion.^19,23^

### 3.2 Analysis of the DEP Assembly Orientations of Customized Trimer Particles

**Figure 9:**
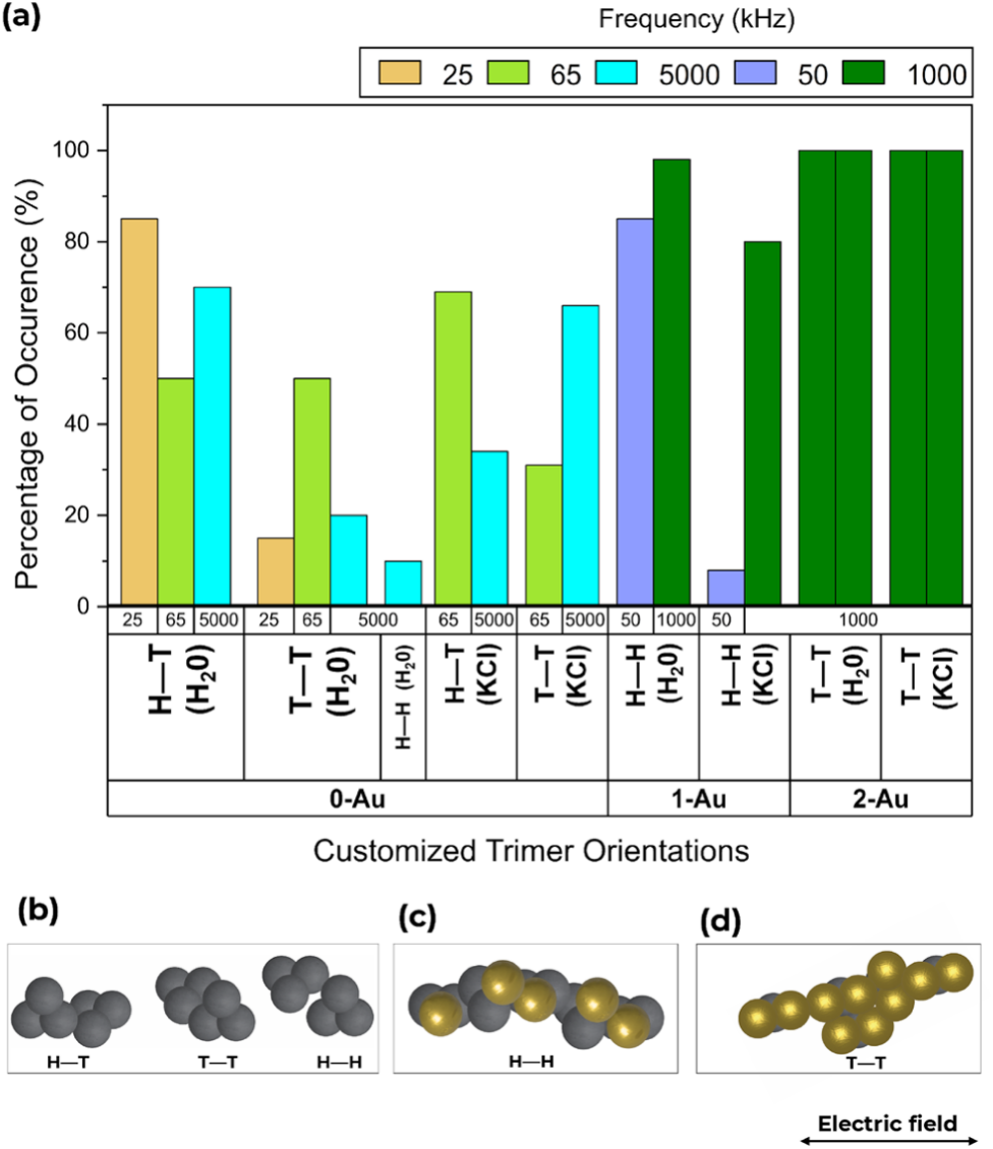
(a) Graph showing the percentage occurrence of different trimer orientations at varying frequencies in deionized water and KCl, respectively, for 100 particles. *H–T* represents “Head-to-Tail”, *H–H* represents “Head-to-Head”, and *T–T* represents “Tail-to-Tail” assembly orientations. (b, c, d) Corresponding schematic representations of select chain assembly orientations for **0-Au**, **1-Au**, and **2-Au** chains under an AC electric field. **0-Au** shows the two major *H–T* and *T–T* orientations with a minor *H–H* orientation, **1-Au** shows the *H–H* orientations, and **2-Au** shows the *T–T* orientations for the gold-coated lobes. Yellow bars, 25 kHz; green bars, 65 kHz; cyan bars, 5 MHz; pastel blue bars, 50 kHz; olive bars, 1 MHz.

Upon assembly in an electric field, different orientation types were observed for the trimer particles. Again, we attribute the “head-to-tail” (H–T), “head-to-head” (H–H), and “tail-to-tail” (T–T) orientations to denote the relationship of a particle with respect to its neighboring particle during the assembly. Figure 9a shows a graph of the percentage of occurrence of particle assemblies with different orientations in different media and with varying frequencies. At a low frequency of 25 kHz, 85% of the linear chains of the **0-Au** particles assume a head-to-tail orientation, while 15% maintained a tail-to-tail orientation in deionized water. However, an observed 50% of **0-Au** particles formed tail-to-tail orientations, and 50% formed head-to-tail orientations at an increased frequency of 65 kHz. The **0-Au** particles in deionized water began experiencing a “crossover frequency” when the frequency was further increased to 5 MHz, causing 70% of the particles to assume a head-to-tail orientation while 20% maintained a tail-to-tail orientation and only 10% showed a head-to-head orientation (Figure 9a,b). Upon variation of the dispersed medium to KCl, a higher frequency of 5 MHz was required to orient the **0-Au** particles, with 66% assuming tail-to-tail orientations while 34% formed head-to-tail orientations. When the frequency was lowered to 65 kHz, a sparse assembly of the particle chains was observed, with 69% assuming head-to-tail orientations while 31% formed tail-to-tail orientations.

Observations for the **1-Au** particle assemblies show that 85% and 98% of the particles assemble with their gold lobes in a head-to-head orientation at 50 kHz and 1 MHz, respectively, in deionized water. Only 80% adopt the same orientation at a frequency of 1 MHz in KCl due to the significantly lower dielectric constant of KCl compared to deionized water. Lowering the frequency to 50 kHz for **1-Au** particles in KCl showed 8% head-to-head orientations. **2-Au** particles all assembled in a tail-to-tail fashion at a frequency of 1 MHz, represented by green bars in Figure 9a, due to the increased metallic moieties as directed by their gold-coated lobes. **3-Au** particle assemblies aligned with the electric field direction at the electrodes of the device due to the high polarizability of the entire gold-coated lobes.

## 4 Conclusions

The introduction of regioselective customization of colloidal trimers by using shape and surface anisotropy has produced a library of colloids with selectively coated gold lobes. The particle assembly process was investigated under a dielectrophoretic field at various time points, varying frequencies of the electric field, and changes in the dispersion medium. The assemblies of **0-Au**, **1-Au**, **2-Au**, and **3-Au** in two distinct media of varying dielectric constants (deionized water and an aqueous potassium chloride solution) displayed a comparative decrease in the overall assembly density of the particles in KCl. This was attributed to the higher conductivity and the significantly lower dielectric constant of KCl in comparison with deionized water. The behavior of particle assemblies in the presence of an electric field can be explained by combining dipolar interparticle interactions with the interactions of dielectric particles from areas of maximum field intensity.^55^ DEP forces can effectively modify particle mobility and interparticle interaction through AC-induced dipoles on the particles. Surfactant-induced separations and residual electrostatic repulsion render vdW forces insufficient to stabilize assemblies without an electric field. Consequently, the vdW forces play a negligible role once the field is removed, enabling on-demand assembly and disassembly of the particles controlled by the applied electric field.^54^ Analysis of particle orientation during assemblies shows that the polymeric **0-Au** particles formed closed stack chain orientations, which eventually cascade into 2-D aggregates with different particle orientations to the neighboring particle. An increase in anisotropy in the trimers by introducing gold-coated lobes to form metallodielectric trimers impacted particle assembly orientation in DEP. The gold-coated lobes dominated the assembly of the metallodielectric particles **1-Au** and **2-Au**, thus orienting the particles in the *z-*direction of the DEP cell, where the electric field is concentrated. **3-Au** particles with complete gold-coated lobes aligned with the field direction at the electrodes, unlike particles **0-Au**, **1-Au**, or **2-Au**, which we attribute to the high polarizability of the metallic character of **3-Au**. This is particularly interesting because it allows for varying frequency and varying applied electric field strength, with limited degrees of assembly tunability not observed for dielectric **0-Au** or metallodielectric **1-Au** and **2-Au**.

The quantitative analysis of the DEP assemblies at a specific time frame shows each particle’s orientation with respect to a neighboring particle during assembly. The rotational order for **0-Au**, with no preferred individual particle orientation, led to more head-to-tail orientations than tail-to-tail orientations at lower frequencies in deionized water. However, at higher frequencies and in aqueous KCl, more tail-to-tail orientations were observed, with a sparse assembly of **0-Au** chains assuming more head-to-tail orientations than tail-to-tail orientations at lower frequencies. Although the gold-coated lobes dominated the assembly of the metallodielectric particles, **1-Au** assemblies show increased head-to-head orientations in deionized water as the frequency increases, but a reduced percentage of head-to-head orientation for **1-Au** assemblies in aqueous KCl and a further reduced fraction of assemblies at lower frequencies. **2-Au** particles all assembled in a tail-to-tail fashion in both media at a frequency of 1 MHz, respectively, and **3-Au** particles all aligned with the electric field direction at the electrodes of the device with no distinct quantifiable orientation. This analysis elucidates the observed assemblies of these complex colloidal particles and distinctively concludes that for trimers without any gold lobes, **0-Au**, a variety of assembly orientations can be achieved with varied parameters. In contrast, **1-Au** assemblies, with mainly head-to-head orientations, can have their percent occurrence easily controlled by switching the media from water to KCl and increasing the frequency of the electric field. **2-Au** particles only show tail-to-tail assemblies, with no head-to-tail assemblies observed.

Multicomponent particle colloidal assemblies have inspired sophisticated design variations, and this progress has facilitated the creation of a wide range of functional materials with tailored properties. The variety of assemblies formed provides insight into the phenomena that occur when complex anisotropic colloidal particles are subjected to an electric field. The approach described provides a toolkit for the fabrication of photonic crystals, waveguides for optical sensors and microcircuits.^5,7,19^ As our understanding of directed colloidal assembly continues to grow, we can expect further advancements in the design, fabrication, and quantification of materials with unprecedented properties.

## 5 Materials and Methods

### 5.1 Materials

3-(Trimethoxysilyl) propyl methacrylate (TPM, > 98.0% (GC), stabilized with BHT) was purchased from TCI Chemicals and used as received. Pluronic F127, dichloromethane (DCM), 2,2-azobis(2-methyl propionitrile) (AIBN), ethylenediamine (≥99.5%), potassium iodide, hexamethyldisilazane (HDMS), poly(vinylpyrrolidone) (PVP, *M*_w_ = 29 K), gold(III) chloride trihydrate (≥99.9% trace metals basis), formaldehyde solution (36.5–38% in H_2_O), and ammonia solution (28% NH_3_ in H_2_O) were purchased from Sigma-Aldrich and used as received. Triton X-100 (TX) (Alfa Aesar) and sodium borohydride (NaBH_4_) (Acros Organics) were used as received without any further purification. 3-Chloro-2-hydroxypropyl methacrylate (CHPMA) (Sigma-Aldrich) was allowed to pass through a column filled with aluminum oxide (basic) to remove the inhibitor. Gold pellets, chromium pellets, and tungsten baskets for metal thermal evaporation were acquired from Ted Pella, Inc. The rotating station was an IKA Loopster digital rotator. Bright-field optical micrographs were acquired by using a Nikon Eclipse TE300 inverted microscope equipped with a Nikon N7000 camera. The ζ potential of the particles was measured using a Malvern Zetasizer Nano. Colloidal particles in the dried state were imaged with a Merlin (Carl Zeiss) field-emission scanning electron microscope (SEM). Particle sizes and energy-dispersive spectroscopy (EDS) were measured from a random selection of ten particles using the Scanning electron microscopy (SEM) analysis tool. Some of the images were digitally postprocessed to improve brightness, contrast, and clarity.

### 5.2 Synthesis of TPM and TPM-Cl Spheres

TPM-Cl spheres were synthesized following a modified reported procedure.^45,56^ In detail, 300 mL of deionized (DI) water was added to a 500 mL Pyrex round-bottom flask with a magnetic stir bar stirred constantly at 400 rpm. Then, 1.8 mL of ammonia solution (28 wt %) was added, and the system was stirred for 15 min, followed by the addition of 3 mL of TPM monomer. The suspension was continuously stirred at 400 rpm for two h at room temperature; a monodisperse micrometer-sized TPM emulsion was produced. To incorporate the chlorine functional group into the TPM emulsion, 1.2 mL of CHPMA was added, allowed to diffuse into the TPM emulsion, and stirred for another 3 h. For bare TPM spheres, this step is skipped. Three milliliters (1.0 wt %) of F127 solution was added and stirred for 1 h. One hundred milligrams of AIBN was added, and the suspension was allowed to stir for another 15 min, after which the oil bath was set to 70 °C for 8 h to polymerize the particles. The reaction was cooled to room temperature, producing TPM-Cl particles, which were then collected and washed with DI water by repeated centrifugation and redispersion before being made into 50 mL of stock suspension (1.4 wt %) for subsequent usage. The particle sizes can be adjusted by varying the amounts of the TPM monomer and ammonia initially added.

### 5.3 Fabrication of Nonlinear Trimer Particles

**0-Au** Trimers: To fabricate the bare TPM trimer particles, a sequential seeded growth synthesis was employed. The already synthesized TPM spheres were used as seeds for the dumbbell-shaped TPM dimer particles (Table S1). Ten milliliters of an aqueous suspension of TPM was prepared in a 24 mL glass vial, followed by the addition of 25 μL of ammonium hydroxide solution (28%, w/w). The colloidal solution was agitated briefly, after which the designated amount of TPM monomer was added. With the vial capped securely, the system was carefully placed on the rotating station (Figure S7A) for 3 h at 16 rpm. Afterward, 500 μL of DCM was added to the mixture and agitated mildly by hand for approximately 3 min. DCM was evaporated out of the mixture in a 60 °C oven within 3 h. The suspension was cooled to room temperature. Then, to incorporate the surfactant onto the surface of the second lobe, 50 μL (1.0 wt %) of an aqueous solution of F127 was added, and the system was again rotated for an additional hour at 16 rpm. Thereafter, 5 mg of AIBN was added to the mixture and placed in a 60 °C oven to polymerize for 12 h. The resulting dimers were retrieved and washed with DI water by centrifugation and redispersion three times before being made into a stock suspension for subsequent usage. Next in the sequence is to fabricate the nonlinear trimer particles. Typically, 1.0 mL stock solution of TPM dimers was dispersed into DI water to make 10 mL of suspension (∼0.04 wt %) in a 24 mL glass vial, followed by the addition of 25 μL of ammonia solution. The colloidal suspension was agitated briefly, after which the designated amount of TPM monomer was added. With the vial capped securely, the system was carefully placed on the rotating station for four h at 16 rpm. Afterward, 500 μL of DCM was added to the mixture, and the mixture was agitated mildly by hand for approximately 3 min. DCM was evaporated out of the mixture in a 60 °C oven over a period of 3 h, after which the suspension was cooled to room temperature. To reduce the surface tension of the additional lobe and to incorporate the surfactant onto the surface of the new lobe, 200 μL of Triton-X (1.0 wt %) and 50 μL (1.0 wt %) of aqueous solution of F127 was added, and the system was again rotated for an additional hour after each addition at 16 rpm. Thereafter, 5 mg of AIBN was added to the mixture and then placed in a 60 °C oven to polymerize for 12 h. The resulting trimers (**0-Au** Trimers) were retrieved and washed with DI water by centrifugation and redispersion three times before being made into a stock suspension for subsequent use.

### 5.4 **1-Cl** TPM Trimers for **1-Au** Trimers

To fabricate the **1-Au** TPM trimer particles, a sequential seeded growth synthesis was employed, followed by a gold customization synthesis. To start with, the already synthesized TPM-Cl spheres were used as seeds for the two-component dimer particles (TPM-Cl dimers). Ten milliliters of aqueous suspension of TPM was prepared in a 24 mL glass vial, followed by the addition of 25 μL of ammonium hydroxide solution (28%, w/w). The colloidal solution was agitated briefly, after which the designated amount of TPM monomer was added. With the vial capped securely, the system was carefully placed on the rotating station for 3 h at 16 rpm. Afterward, 500 μL of DCM was added to the mixture and agitated mildly by hand for approximately 3 min. DCM was evaporated out of the mixture in a 60 °C oven within 3 h. The suspension was cooled to room temperature. To incorporate the surfactant onto the surface of the second lobe, 50 μL (1.0 wt %) aqueous solution of F127 was added, and the system was again rotated for an additional 1 h at 16 rpm. Thereafter, 5 mg of AIBN was added to the mixture and then placed in a 60 °C oven to polymerize for 12 h. The resulting dimers were retrieved and washed with DI water by centrifugation and redispersion three times before being made into a stock suspension for subsequent usage. Next in the sequence is to fabricate the nonlinear trimer particles. Typically, 1.0 mL stock solution of TPM-Cl dimers was dispersed into DI water to make 10 mL of suspension (∼0.04 wt %) in a 24 mL glass vial, followed by the addition of 25 μL of ammonia solution. The colloidal suspension was agitated briefly, after which the designated amount of TPM monomer was added. With the vial capped securely, the system was carefully placed on the rotating station for 4 h at 16 rpm. Afterward, 500 μL of DCM was added to the mixture and agitated mildly by hand for approximately 3 min. DCM was evaporated out of the mixture in a 60 °C oven within 3 h, after which the suspension was cooled to room temperature. To reduce the surface tension of the additional lobe and to incorporate the surfactant onto the surface of the new lobe, 200 μL of Triton-X (1.0 wt %) and 50 μL (1.0 wt %) aqueous solution of F127 were added, and the system was again rotated for an additional 1 h after each addition at 16 rpm. Thereafter, 5 mg of AIBN was added to the mixture and then placed in a 60 °C oven to polymerize for 12 h. The resulting trimers (**1-Au** Trimers) were retrieved and washed with DI water by centrifugation and redispersion three times before being made into a stock suspension for subsequent use.

### 5.5 Site-Specific Gold Coating of Nonlinear Trimer Particles

Using a modified method,^52^ chlorine groups on the specific lobe of the TPM-Cl particle surfaces were first converted to amine groups, which facilitated the site-specific gold coating of the lobe (Figure S3). Typically, 2 mL of the multicomponent particles’ suspension (0.5% w/w) containing 1.0% F127 was treated with 300 μL of ethylenediamine and a trace amount of potassium iodide (KI). After 4 h of reaction at 70 °C, amine-functionalized particles were obtained. There were two steps in the gold coating process. In the first step, 120 μL of an aqueous chloroauric acid (HAuCl_4_) solution (4 mg/mL) was added to the amino-functionalized particles, and the mixture was vortexed for 3 min. Thereafter, the particle suspension was washed three times with DI water, and designated sodium borohydride (NaBH_4_) was added to reduce Au^3+^, creating gold nanoparticles on the particle surface. After gently shaking for 5 min, the particle suspension was washed with DI water three consecutive times and dispersed in 0.5 mL of 0.3% poly(vinylpyrrolidone) (PVP, 29 K) aqueous solution. PVP, as a surfactant, is expected to reduce the surface strain difference between the two regions, thus changing the wetting degree. The gold nanoparticles served as seeds and allowed the growth of the uniform gold layer on the site-specific lobe. For this purpose, freshly made gold hydroxide (Au(OH)_3_, 600 μL) (by dissolving 200 mg of K_2_CO_3_ in 20 mL of a 4 mg/mL HAuCl_4_ aqueous solution and stirring overnight in the dark to obtain a colorless, basic solution) and formaldehyde (HCHO, 67.5 μL) were incorporated into the system. The solution was transferred to a 3 mL glass vial, and with the cap securely fastened, the system was gently rotated on the rotating station overnight at 6 rpm to produce a relatively smooth gold coating with even thickness.

## Supporting Information Available

Movie S1: DEP assembly of **0-Au** trimer particles in deionized water at *f* = 5 MHz and constant electric field intensity of *E* = 200 Vcm^–1^. The particles were assembled over a period of 2 h (MP4)
Movie S2: DEP assembly of **1-Au** trimer particles in deionized water at *f* = 1 MHz and constant electric field intensity of *E* = 200 Vcm^–1^. The particles were assembled over a period of 2 h (MP4)
Additional experimental details, materials, and methods (PDF)
Movie S3: DEP assembly of **2-Au** trimer particles in deionized water at *f* = 1 MHz and constant electric field intensity of *E* = 200 Vcm^–1^. The particles were assembled over a period of 2 h (MP4)
Movie S4: DEP assembly of **3-Au** trimer particles in deionized water at *f* = 1 MHz and constant electric field intensity of *E* = 200 Vcm^–1^ (MP4)
