# Supplementary material for: Dielectrophoretic Assembly of Customized Colloidal Trimers
Source: ACS Nanosci Au. 2025 Mar 12;5(2):100–10. doi: 10.1021/acsnanoscienceau.5c00007 (PMC12006858; doi:10.1021/acsnanoscienceau.5c00007)
Supplement: Supplementary file 3 — ng5c00007_si_003.pdf [file ng5c00007_si_003.pdf]

Dielectrophoretic Assembly of Customized Colloidal Trimers

Supporting Information: Additional experimental details, methods, characterizations and assemblies.

Samira Munkaila, Kevin J. Torres, Jennifer Wang and Marcus Weck*

Molecular Design Institute and Department of Chemistry, New York University, New York, NY 10003, United States

* Corresponding author's email address: marcus.weck@nyu.edu

Table of Contents

Experimental Section……………..………………………..……..…………….……….…..………....2

Table S1. Synthetic summary of fabricating each non-linear trimer particle type ……..................4

Figure S1. SEM images of TPM spheres and corresponding TPM-Au spheres ……………….……...5

Figure S2. SEM images of linear-shaped trimers showing the effect of hydrolysis time ……...……6

Figure S3. Detailed schematic of trimer particle synthesis and customization…………..……...…6

Figure S4. Effect of $NaBH_4$ on trimer customization with gold coated nanoparticles…………...…7

Figure S5. Optical micrographs of **0-Au** assembled in aqueous KCl…….………………………7

Figure S6. Zeta potentials of the customized trimers in deionized water and aqueous KCl…..…..8

Figure S7. Picture of the rotating station at the Weck lab and Schematic illustrations of the DEP device…………………………………………………………………………………….....8

Supporting References…...…………………………………….………………..................... 9

Other supporting materials for this manuscript include the following:

Movies S1 to S4

Experimental Section

Fabrication of non-linear trimer particles.

2-Cl TPM trimers for 2-Au Trimers: To fabricate the **2-Au** TPM trimer particles, a sequential seeded growth synthesis was employed followed by a gold customization synthesis. To start with, the already synthesized TPM-Cl spheres were used as seeds for the two-component dimer particles (TPM-Cl dimers). 10 mL of an aqueous suspension of TPM was prepared in a 24 mL glass vial, followed by the addition of 25 μL ammonium hydroxide solution (28%, w/w). The colloidal solution was agitated briefly after which the designated amount of TPM monomer was added. With the vial capped securely, the system was carefully placed on the rotating station for three hours at 16 rpm. Afterward, 500 μL DCM was added to the mixture and the mixture was agitated mildly by hand for approximately three minutes. DCM was evaporated out of the mixture in a 60°C oven within three hours. The suspension was cooled to room temperature followed by addition of 10 μL CHPMA to allow the methacrylate to diffuse into the TPM emulsion. The system was again rotated for an additional hour at 16 rpm to incorporate the chlorine functional group into the second TPM dimer lobe. To incorporate the surfactant onto the surface of the second lobe, 50 μL (1.0 wt.%) aqueous solution of F127 was added, and the system was again rotated for an additional hour at 16 rpm. Thereafter, 5 mg AIBN was added to the mixture and then placed in a 60°C oven to polymerize for 12 hours. The resulting dimers were retrieved and washed with DI water by centrifugation and re-dispersion, three times before being made into a stock suspension for subsequent usage (Figure S3)

Next in the sequence is to fabricate the non-linear trimer particles. Typically, 1.0 mL stock solution of TPM-Cl dimers was dispersed into DI water to make a 10 mL suspension (~0.04 wt%) in a 24 mL glass vial followed by the addition of 25 μL ammonia solution. The colloidal suspension was agitated briefly, after which the designated amount of TPM monomer was added. With the vial capped securely, the system was carefully placed on the rotating station for four hours at 16 rpm. Afterward, 500 μL DCM was added to the mixture and the mixture was agitated mildly by hand for approximately three minutes. DCM was evaporated out of the mixture in a 60°C oven over a period of three hours after which the suspension was cooled to room temperature. To reduce the surface tension of the additional lobe and to incorporate the surfactant onto the surface of the new lobe, 200 μL Triton-X (1.0 wt.%) and 50 μL (1.0 wt.%) of an aqueous solution of F127 were added, and the system was again rotated for an additional hour after each addition at 16 rpm. Thereafter, 5 mg AIBN was added to the mixture and then placed in a 60°C oven to polymerize for 12 hours. The resulting trimers (**2-Au** Trimers) were retrieved and washed with DI water by centrifugation and re-dispersion, three times before being made into a stock suspension for subsequent use.

3-Cl TPM trimers for 3-Au Trimers: To fabricate the **3-Au** TPM trimer particles, a sequential seeded growth synthesis was employed followed by a gold customization synthesis. To start with, the already synthesized TPM-Cl spheres were used as seeds for the two-component dimer particles (TPM-Cl dimers). 10 mL of an aqueous suspension of TPM was prepared in a 24 mL glass vial, followed by the addition of 25 μL ammonium hydroxide solution (28%, w/w). The colloidal solution was agitated briefly, after which the designated amount of TPM monomer was added. With the vial capped securely, the system was carefully placed on the rotating station for three hours at 16 rpm. Afterward, 500 μL DCM was added to the mixture and the mixture was agitated mildly by hand for approximately three minutes. DCM was evaporated from the mixture in a 60°C oven over a period of three hours. The suspension was cooled to room temperature, followed by the addition

of 10 µL CHPMA to allow the methacrylate to diffuse into the TPM emulsion. The system was again rotated for an additional hour at 16 rpm to incorporate the chlorine functional group into the second TPM dimer lobe. To incorporate the surfactant onto the surface of the second lobe, 50 µL (1.0 wt.%) aqueous solution of F127 was added, and the system was again rotated for an additional hour at 16 rpm. Thereafter, 5 mg AIBN was added to the mixture and then placed in a 60°C oven to polymerize for 12 hours. The resulting dimers were retrieved and washed with DI water by centrifugation and re-dispersion three times before being made into a stock suspension for subsequent usage.

A similar protocol to that described above was used to synthesize the 3-Au non-linear trimer particles from the synthesized dimers. The resulting particles were retrieved and washed with DI water by centrifugation and re-dispersion three times before being made into a stock suspension for subsequent use. Table S1 shows a detailed recipe for fabricating each polymeric non-linear trimer particle type.

Density gradient centrifugation.

Further purification steps were taken to obtain monodisperse and symmetrical respective trimer particles. One was to wash the particles with DI water, centrifuge, and re-disperse. The other was to employ density gradient centrifugation techniques. A 20-40% w/w linear gradient of a glycerol-water mixture was prepared by a gradient maker (Gradient Master 108, Bio-Comp Instruments), and 0.5 mL of the site-specific gold coated multicomponent particles solution was added to the upper surface of the 12 mL of gradient solution. Various parameters ranging from 800 rpm for 12 minutes to 1500 rpm for 15 minutes were used to selectively separate the multicomponent particles ahead of DEP assembly experiments.

Zeta Potential.

The zeta potentials of each category of trimer particles for the different dispersion media and conductivities were measured with a commercial zeta potential analyzer. The electrical potential at the electrical double layer of particles measures particle stability with a zeta potential value of ±25 mV, showing moderate stability and ±60 mV, depicting excellent stability of the particles.[1] In water, the particles measured an average zeta potential of -41.57 mV, whereas, in KCl, they measured an average of -18.99 mV, indicating that water is preferred to KCl for particle stability.

DEP-induced self-assembly of the non-linear trimer particles.

The device was assembled according to a method previously described.[2] In a typical setup as shown in Figure S7-B, a 10 nm thick chromium layer and a 30 nm thick gold layer were sequentially deposited onto a glass slide of variable thickness (typically 130 to 160 µm thickness) via thermal evaporation (BAL-TEC MED 020). A rectangular glass tube, 400 µm wide, was used in the middle of the intended channel as a mask to create and pattern two parallel electrodes in a coplanar arrangement. After the process of thermal evaporation was complete, the glass tube was removed, and then pre-treated with oxygen plasma for three minutes and exposed to HMDS vapor to make

its surface hydrophobic. Before usage, the pre-treated glass side was rinsed with a 1 mL (1.0 wt.%) F127 solution to prevent particles from sticking to the intended glass channel. A channel was then constructed by affixing two pieces of adhesive tape, about 120 µm thick, along the edges of the electrodes as spacers. Then, a glass coverslip was attached to the top surfaces of the two spacers with UV-curable, thiolene-based adhesive (NOA 81, Norland Products). Copper wire (500 µm diameter) was attached to each electrode with copper tape and connected to an AC power source (BK Precision 4007B). The purified particles were suspended in DI water and injected into the channel of the DEP device. The ends of the channel were sealed with wax to prevent the evaporation of the solution and ambient interferences. The particle suspension was allowed to settle for two minutes before applying a square wave AC electric field across the electrodes. A function generator (4007DDS, BK Precision) was connected to the input side of a voltage amplifier (5X, 10X, and 20X). The two electric leads of the dielectrophoretic cell were connected to the output side of the amplifier with a 1 µF capacitor installed on one of the leads to eliminate residual parasitic direct current in the circuit. The AC electric field used here was applied at frequencies between 10 kHz and 5 MHz, with field strengths of 75-300 V/cm. The dynamics of both locomotion and assembly of the particles were observed on a Nikon Eclipse TE300 inverted microscope equipped with a Nikon N7000 camera.

Table S1. Summary of seed dimer particle, volume of TPM monomer, and CHPMA used to fabricate each non-linear trimer particle type.

Non-linear trimer particle	Seeds for dimers	Seeds for trimers	TPM monomer/µL	CHPMA/µL
**0-Au**	TPM spheres	TPM dimers	30	0
**1-Au**	TPM-Cl spheres	1-Cl TPM dimers	30	0
**2-Au**	TPM-Cl spheres	2-Cl TPM dimers	30	10
**3-Au**	TPM-Cl spheres	3-Cl TPM dimers	30	10

Supplementary Figures

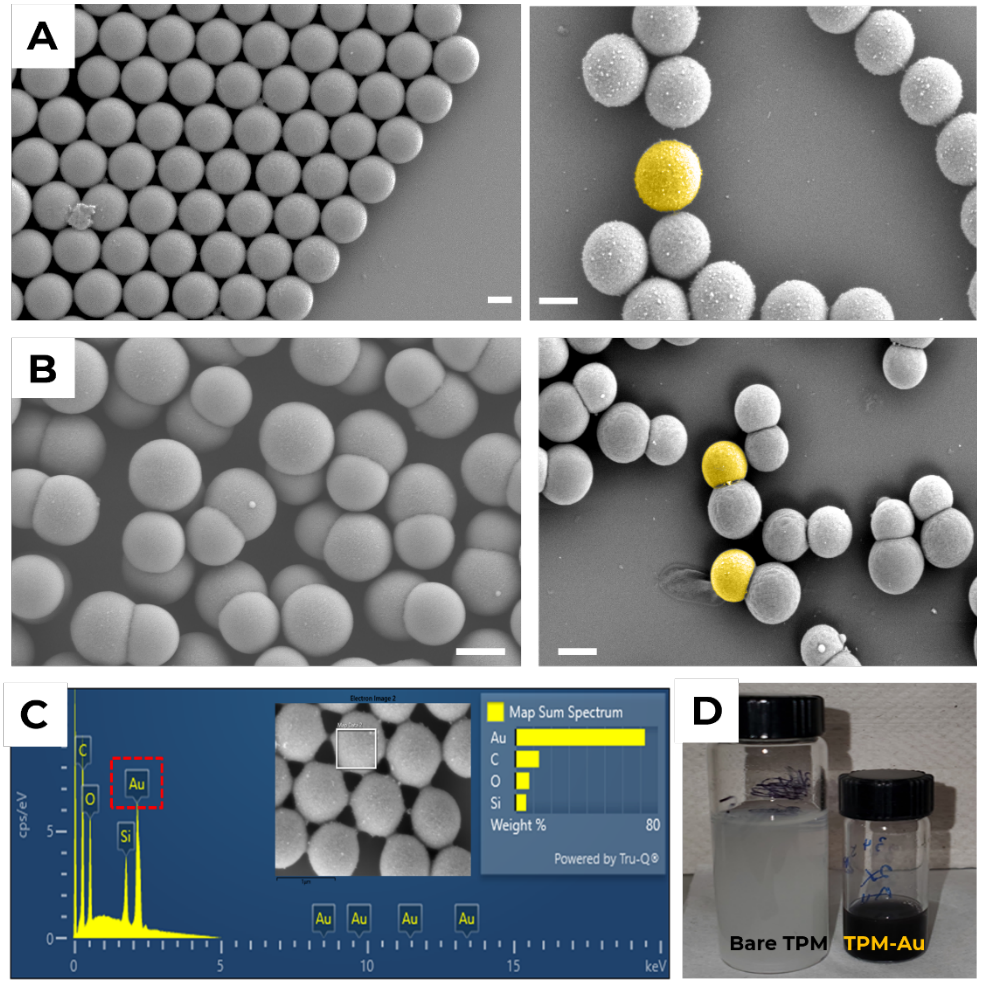

Figure S1. Scanning electron microscopy (SEM) images of (A) TPM-Cl spheres and corresponding TPM-Au spheres with diameter of 1.56 ± 0.08 μm, diameter values of particles were measured with SEM analysis tool for ten particles; (B) dimers with corresponding TPM-Au dimers with one lobe coated with gold nanoparticles. The gold coated component has a brighter contrast and has been falsely colored for clarity. (C) Energy dispersive spectrum of gold coated TPM spheres. Insert shows the SEM micrograph of the analyzed portion of the TPM spheres. (D) Actual colloidal particles of bare TPM spheres (cloudy solution) and gold coated TPM (TPM-Au) in glass vials. Scale bars, 1 μm.

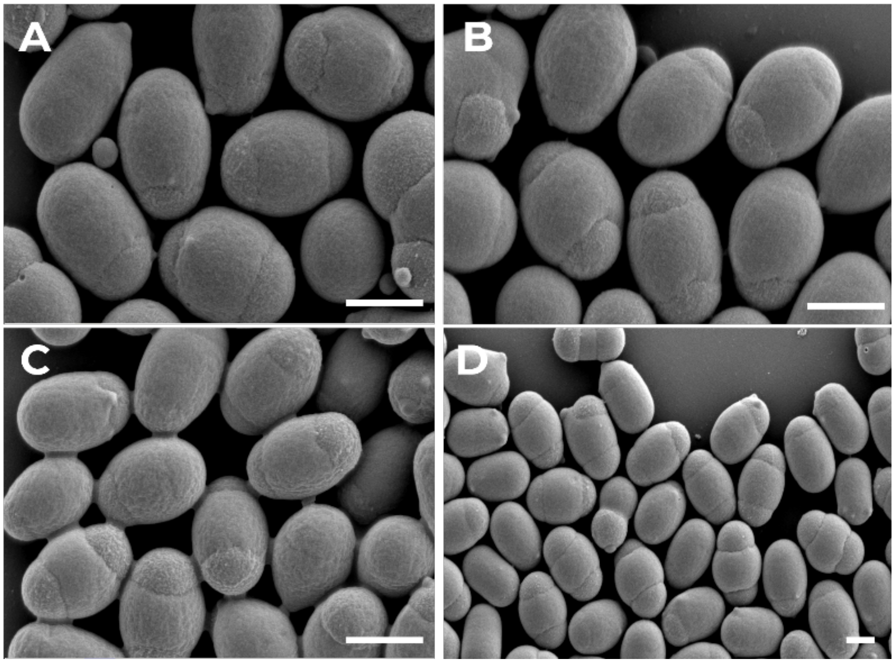

Figure S2. Scanning electron microscopy (SEM) images of linear-shaped trimers before gold coating; A longer hydrolysis time promotes the spread of TPM on seed surfaces. (A, B) has a comparatively higher monomer concentration than (C, D). Scale bars, 1μm.

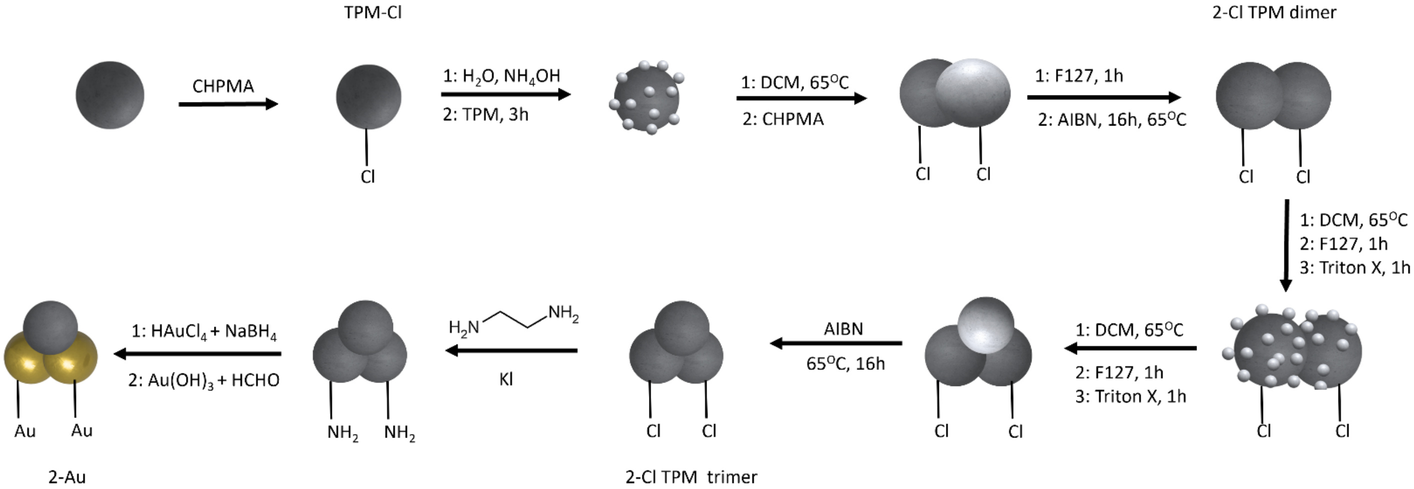

Figure S3. Detailed schematic of **2-Au** trimer particle synthesis and customization.

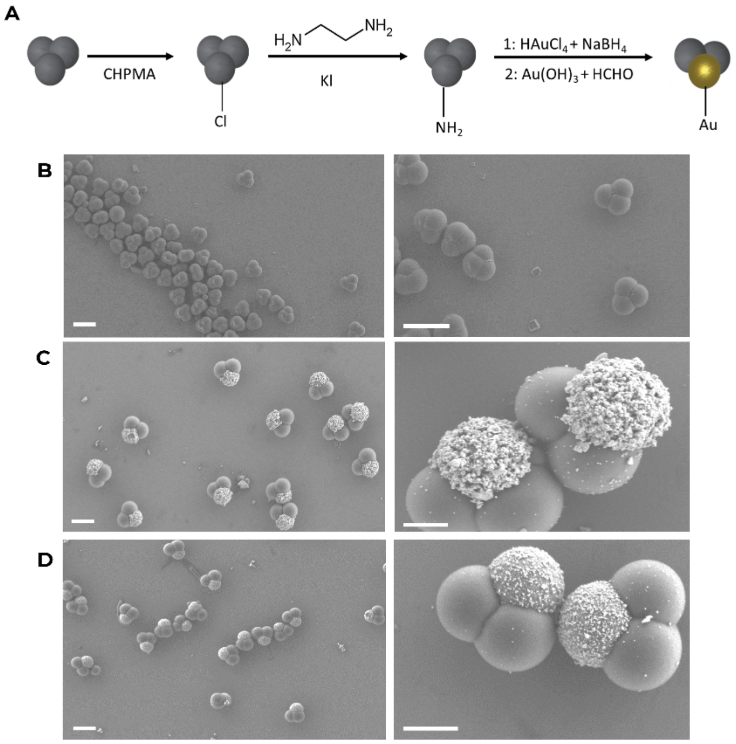

Figure S4. Summary of trimer customization with gold coated nanoparticles. (A) Schematic showing the site-specific lobe customization of the **1-Au** trimer particle (B) SEM images of **0-Au** trimer particles. (C, D) SEM images of **1-Au** trimer particles with increasing concentrations of $NaBH_4$, respectively. Scale bars = 1µm.

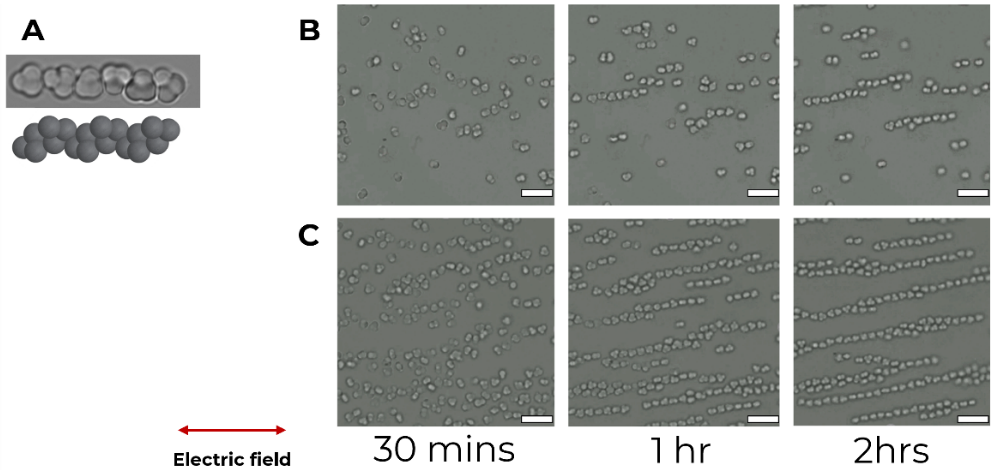

Figure S5. Optical micrographs of **0-Au** in KCl (0.15 mM, $\mathcal{E}$= 49), under DEP force with increasing frequency and constant electric field intensity, E = 200 Vcm^{-1}. (A) Excerpt of **0-Au** trimer assembly with a side-by-side packing orientation and corresponding schematic illustration of packing at f = 65 kHz (B) Trimer particles assembly orientation at f = 65 kHz (C) Trimer particles assembly orientation at f = 5 MHz. Scale bars = 10 µm. Red arrow indicates the direction of the electric field.

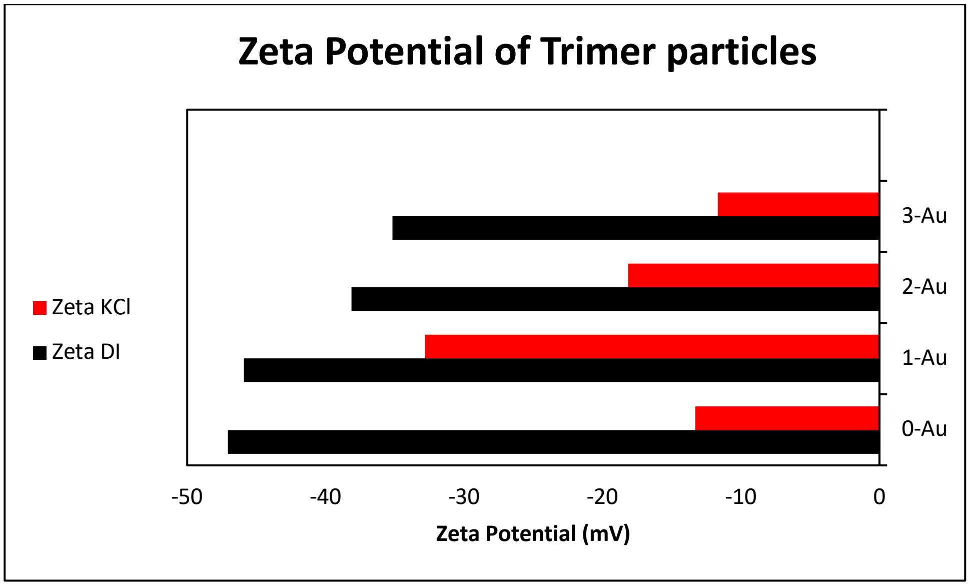

Figure S6. Zeta potential of the customized trimers in deionized water ($\mathcal{E}$=78.5) and KCl (0.15 mM, $\mathcal{E}$= 49), at pH = 7 and 25°C.

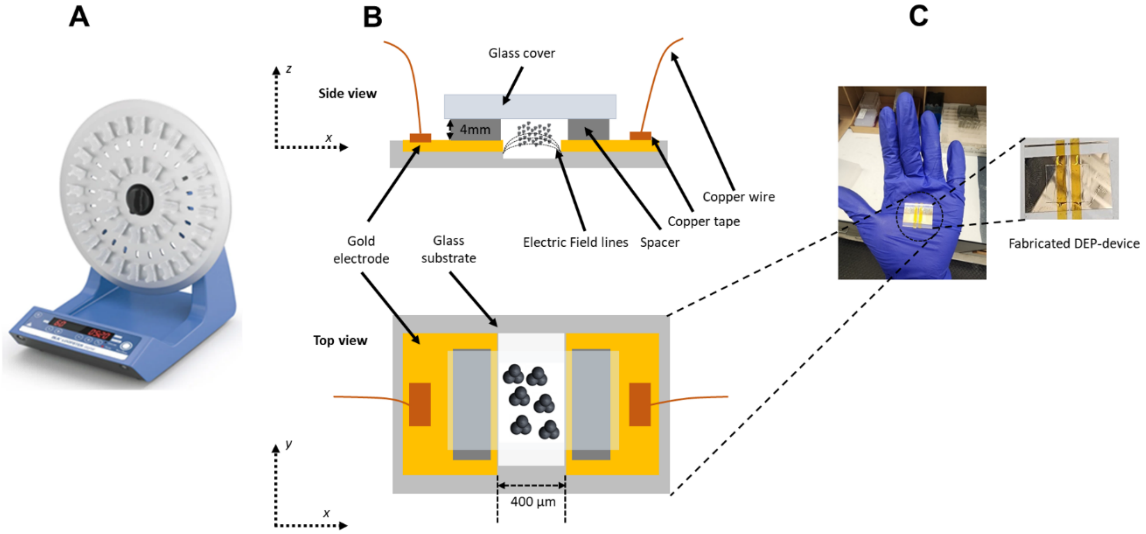

Figure S7. (A) Picture of the rotating station. The particle suspensions in the glass vials were attached as shown, and the rotating speeds were adjusted as described in the experimental section. (B) Schematic illustrations for the top and side view of the DEP device. (C) The actual DEP device is held in the palm of a gloved hand.

Supporting References

1. H. Zhou, M. A. Preston, R. D. Tilton and L. R. White, *J. Colloid Inter. Sci.* **2005**, 285, 845-856.
2. F. Dong, S. Munkaila, V. Grebe, M. Weck and M. D. Ward, *Soft Matter*, **2022,** 18, 7975-7980.
